# Prevalence and genotyping of *Toxoplasma gondii* in stray cats in Mashhad area, Iran

**DOI:** 10.1186/s12917-019-2176-2

**Published:** 2019-12-21

**Authors:** Majid Khodaverdi, Gholamreza Razmi

**Affiliations:** Department of Pathobiology, Faculty of Veterinary Medicine, Ferdowsi University of Mashhad, P.O. Box: 91775-1793, Mashhad, Iran

**Keywords:** *Toxoplasma gondii*, Cats, Genotype, Mice bioassay, PCR-RLFP

## Abstract

**Background:**

Cats as a definitive host have an important role in the epidemiology of toxoplasmosis in humans and animals. The aim of the study was to determine the frequency of *Toxoplasma gondii* infection and isolate and identify the genotypes of *T. gondii* in stray cats in the Mashhad suburb.

**Methods:**

From April 2016 to August 2017, 175 fecal samples from stray cats and 31 brain samples from cats killed in driving accidents were collected. The fecal samples were examined by fecal flotation technique and *T. gondii*-specific PCR. The brain samples were investigated by *T. gondii*-specific PCR and consequently examined by mice bioassay. The DNA of *T. gondii* isolated was genotyped using SAG1, SAG2, SAG3, BTUB and GRA6 as PCR-restriction fragment length polymorphism (PCR-RFLP) markers.

**Results:**

In the present study, *Toxoplasma*-like oocysts were microscopically observed in 2.2% (4/175) fecal samples. The presence of *Toxoplasma* oocysts was confirmed in one microscopy-positive sample by PCR. In addition, T*. gondii* DNA was detected in 4% (7/175) microscopy-negative samples using PCR. *T. gondii* was isolated from one brain PCR-positive sample by mice bioassay. The isolate was avirulent and many *T. gondii* cysts were observed in mice brain. The isolate was successfully genotyped by PCR-RLFP analysis. The isolated genotyped was type II. Besides, eight *Toxoplasma-*positive fecal samples contained insufficient DNA and only amplified at SAG-3 locus in PCR. These samples were also showed type II pattern at this locus.

**Conclusions:**

Parasitological and molecular results showed low frequency of *Toxoplasma* infection in the stray cats, and identified the genotype of *T. gondii* isolate as type II, for the first time in Mashhad area, Khorasan Razavi Province.

## Background

Toxoplasmosis is considered an important zoonotic disease caused by *T. gondii,* an obligate intracellular protozoan [1]. Sexual stage develops only in cat and other felids as the definitive hosts that excrete heavy walled oocysts in feces. It typically occurs in humans and other warm-blooded animals as intermediated hosts tachyzoites are formed first, followed by the formation of tissue cysts. *T. gondii* infection is also transmitted by different routes in humans and animals. Humans acquire *Toxoplasma* infection by eating undercooked or raw meat containing viable tissue cysts, or by direct ingesting of sporulated oocysts and or by congenital route [1, 2].

A large proportion of *T. gondii* infection is asymptomatic in humans, but may lead to acute and fatal toxoplasmosis in immunocompromised patients [3]. Congenital toxoplasmosis can cause abortion, stillbirths or fetal death [4]. The severity of toxoplasmosis is associated with genetics and immunity of host and *Toxoplasma* strains [1].

Based on the virulence levels of *Toxoplasma* strains in outbred mice, strains were classified into three genotypes: I, II and III [5]. Multilocus PCR-restriction fragment length polymorphism (PCR-RFLP), microsatellite DNA analysis and multilocus DNA sequence typing of intron methods have been used to determine the *T. gondii* genotype in many studies [6, 7]. More genotyping studies used multilocus PCR-RLFP analysis of five to ten markers. Among these markers, SAG1, SAG2, SAG3, BTUB, GRA6 could clearly differentiate different genotypes by using nested PCR reactions followed by endonuclease digestion [8–11]. So far, many *Toxoplasma* types were identified that were genetically different with classical types and some have been categorized under unclonal genotypes [9, 12]. An infected cat as the definitive host may shed 1 billion oocysts during primary infection and have the main role in the epidemiology of toxoplasmosis [1] .Many seroepidemiological studies have been performed on *T. gondii* infection in humans and animals in Iran [13]. The overall seroprevalence of *Toxoplasma* infection was estimated to be 22–86% in cats [13]. Despite a high seroprevalence of *T. gondii* in cats in Iran, there are few studies on genetic characterization of T. *gondii* isolates in cats. The present study was designed to determine the occurrence of *T. gondii* in cat feces and to isolate and identify *T. gondii* genotype by using mouse bioassay and PCR-RFLP.

## Results

A total of 175 fecal samples, low number *Toxoplasma-*like oocysts with a diameter 9–12 μm, were microscopically observed in 2.2% (4/175) of fecal samples, whereas, *T. gondii* DNA was detected in 4/5% (8/175) of fecal samples by nested-PCR. One infected fecal sample with *Toxoplasma*-like oocysts was positive only by nested-PCR. No significant statistical differences were identified between the prevalence of *T. gondii* infection in different age and gender groups of stray cats (Table 1) (*p* > 0.05). The DNA of *T. gondii* was detected in 3.2% (1/31) of the brain samples and 6.8% (2/31) fecal samples of dead cats by PCR. All brain samples were examined by mice bioassay, *T. gondii* was isolated only from the PCR-positive brain sample. Poor agreement was observed between parasitological and PCR methods (Kappa = 0. 0.127).

**Table 1:** Results of fecal flotation technique and PCR examination of feces of stray cats in Mashhad area

Variable	Fecal flotation technique		PCR of Feces		Total
	Negative No	Positive No (%)	Negative No	Positive No (%)	
Gender					
Male	58	3(4.9)	58	3(4.9)	61
Female	113	1(0.9)	109	5(4.8)	114
Age (year)					
<1	32	1(3)	32	1 (3)	33
1-3	53	1(1.8)	53	1(1.5)	54
> 3	86	2(2.2)	82	6 (6.8)	88
Total	171	4 (2.2)^a^	167	8 (4.5)^a^	175

^a^only one sample was positive both parasitology and PCR

Many *T. gondii* tissue cysts were microscopically observed at 6 wk. PI in the brain smears of inoculated mice. The size of cysts range was 7–22 μm. The course of infection was without symptoms in all infected mice, thus indicating the isolation of an avirulent (murine) strain. The five multilocus PCR-RFLP analyses revealed that the isolate of brain mice gave restriction digest patterns consistent with infection with type II. Eight *Toxoplasm*a-positive fecal samples were also genotyped using PCR-RLFP analysis. These samples contained insufficient DNA *Toxoplasma* genotyping and only amplified at SAG-3 locus in PCR. The type II pattern was also observed at this marker. The amplified B1 genes of the *Toxoplasma* isolate was sequenced and deposited in GenBank (NCBI) under accession no of MH673033.

## Discussion

In the current study, *T. gondii*-like oocysts were microscopically detected in 2.2% (4/175) of fecal samples. The presence of *Toxoplasma* oocysts was confirmed in one microscopy-positive sample by PCR. Other samples may be infected with other *T. gondii*-like oocysts such as *Hammondia* spp. In the present study, no *T. gondii*-like oocysts were detected in 4% (7/175) of PCR positive samples. This result may be due to consumption of meat contaminated with *Toxoplasma* cysts or due to the small number of *Toxoplasma* oocysts in fecal specimens which are difficult to determine by fecal examination. Similar to our results, a low prevalence of oocysts shedding in cats was determined at 1.2% in Iran [14], at 2.3% in Italy [15], at 0.3% in Japan [16], at 0.14% in Germany [17], at 0.4% in Switzerland [18], at 4.7% in South Korea [19], at 0.9 in the USA [20], at 0.8% in Thailand [21], at 0.76% in Finland [22] by microscopy and PCR methods. In contrast to serological studies, examining feces did not show any information about age and gender as risk factors, due to the low prevalence of oocyst shedding by the cats [13, 23].

*Toxoplasma gondii* was isolated from the brain of a PCR-positive stray cat by mice bioassay. All inoculated mice survived and developed antibodies against *T. gondii* until sacrifice time, thus indicating the isolate belongs to avirulent strain [5, 24, 25]. In the previous study, a non-virulent strain was isolated from aborted ovine fetuses in Mashhad, Iran, by bioassay method [26].

The PCR-RFLP assay at five markers revealed that the avirulent isolate in this study belongs to type II clonal lineage. These five markers have been successfully used in *T. gondii* genotypes in cats in previous studies and proved to identify the genotype of isolates in cats [27–29]. Although, ten genetic markers allow isolates with high resolution [30]. In Iran, types II and III isolates have been detected by RLFP analysis at GRA6 locus [31, 32] and type I and III by RLFP analysis at SAG 2 locus in stray cats [33]. In addition, type II has been detected in humans, sheep and birds by PCR- RLFP assay at GRA6 and in wild rats by SAG 1 locus [34, 35]. However, a single marker was used to genotype *Toxoplasma* in these studies but it does not allow identification of nonclonal strains, and to determine more precisely the presence of polymorphisms in the population [7, 36]. Our result also agreed with similar studies in other countries that type II was detected in stray cats using PCR-RLFP [20, 23, 29, 37–41]. In Europe and North America, type II is the most prevalent strain isolated from both humans and animals [6, 42].

## Conclusions

Based on our results, the presence of *T. gondii* oocysts was identified in one fecal sample of stray cats. In addition, *T. gondii* type II genotype was identified for the first time in stray cats in Mashhad area. Further, similar studies with more markers are required to provide wider insight about the different *Toxoplasma* strains in cats in different parts of Iran.

## Methods

### Research location

**Fig. 1:**
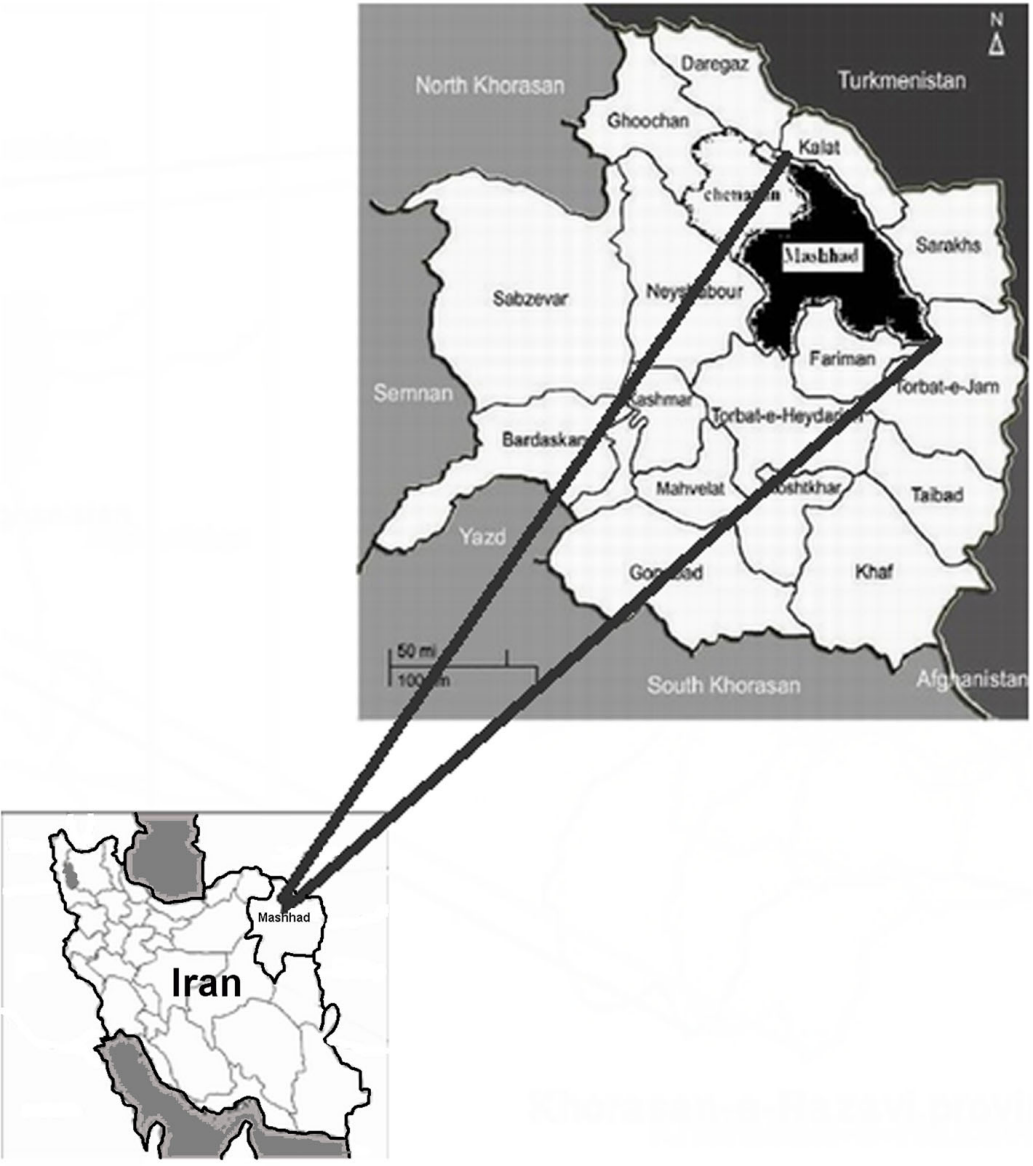
The map of sampling location in Khorasan Razavi province, Iran. The sources of the map of Khorasan province was from the our previous study at http://ijpa.tums.ac.ir/index.php/ijpa/article/view/284 and the map of Iran at http://en.wikipedia.org/wiki/File:Locator_map_Iran_Razavi_Khorasan

The study was performed in Mashhad area as the center city of Khorasan Razavi province from April 2016 to August 2017. The city is located at 36.20° North latitude and 59.35° East longitude, in the valley of the Kashafrud River near Turkmenistan, between the two mountain ranges of Binalood and Hezar Masjed Mountains. The city benefits from the proximity of the mountains, having cold winters, pleasant springs, and mild summers (Fig. 1).

### Sampling

One hundred seventy- five stray cats were trapped from different areas of the Mashhad in with the help of local municipality. Furthermore, Thirty-one samples were from killed stray cats during driving accidents. The trapped cats and carcasses were transferred to the diagnostic laboratory of the parasitology department for laboratory examination. They were sedated by premedication with intramuscular (IM) Ketamine hydrochloride (6 mg/kg). The approximate age of the cat was determined by teeth examination. All of the adult teeth are in place by 6 months of age, and the growth is no longer useful in determining a cat’s age. In older cats, the amount of staining, or tartar, on a cat’s teeth is also an indicator of age. Then, the data related to their age, the sex were recorded and collected feces from each cat. The killed cats were necropsied and feces and the brain were collected. Collected fecal and brain samples were kept in a refrigerator at 4 °C for further examination. The trapped cats were released after sampling with the help of the Mashhad municipality.

### Parasitological method

Feces (1 g) of each animal were emulsified in sucrose solution (specific gravity 1.203), filtered through gauze, and centrifuged in a 15 mL tube at 400 g for 10 min. A drop of the float from the meniscus was examined microscopically at × 400 magnifications for the presence of *T. gondii* oocysts [1]. The size of oocysts was measured by a calibrated ocular micrometer (Zeiss Company, Germany).

### Nested-PCR amplification

Oocysts of fecal samples were repeatedly washed in PBS and homogenized by grinding with 0.5 mm glass beads for 30 min. DNA of homogenized oocysts, fecal and brain samples were extracted by a commercial kit (Molecular and Biological Transmission Systems (MBST), Tehran, Iran) as per manufacturer’s recommendations. *T. gondii* B1 gene PCR amplification was carried out using a nested-PCR, as previously described by Burg et al. [43]. Amplification in 25 μL reaction volumes (Accupower PCR premix kit, Bioneer®, South Korea) in the first reaction contained: 250 μM of each dNTP, 10 mM Tris-HCl pH 9.0, 30 mM KCl and 2 mM MgCl2, 1 U Taq DNA polymerase and 10 pmol of each PCR primer (Denazist, Mashhad, Iran). Then 1 μL of DNA template (250–500 ng) was added to each reaction and the remaining 25 μL reaction volume was filled with sterile distilled water.

After 3 min of initial denaturation at 94 °C, 38 cycles of amplification (each cycle: 1 min at 94 °C, 1 min at 50 °C, and 1 min at 72 °C) and a final extension step for 7 min at 72 °C were performed in an automated thermocycler (MJ Mini Thermal Cycler, Bio-Rad Co, USA). The PCR products were visualized by electrophoresis on a 1.5% agarose gel. One μL of the diluted (1,10) each reaction is then used in the second reaction in the same mixture and cycling condition, except for the annealing temperature, which was 52 °C; the number of cycles was 30. The presence of specific bands of 193 bp in primary PCR and 96 bp in nested -PCR on agarose gel was considered a positive sample [43]. Distilled water were used as a negative control and *T. gondii* strains (RH) was used as positive controls.

### Isolation of *T*.*gondii*

The tissue homogenates were prepared from the brain tissue of the cats as the method described by Dubey [1]. Briefly, 100 g of brain samples were homogenized in 0.5 L of normal saline (0.85%) with penicillin 100 IU/mL and streptomycin 1 mg/mL by the electrical mixture. The tissue homogenate was strained through 2 layers of gauze to remove coarse material. The homogenate was kept at room temperature for 3 hours and centrifuged at 1.500 g for 5 min. The homogenate (0.5 mL per each mouse) inoculated subcutaneously to Swiss Webster mice (Razi Vaccine& serum research institute, Mashhad, Iran). None inoculated mice were shown clinical signs. Six weeks after inoculation, blood samples were collected from the tail of mice. Serum samples were separated and analyzed for the presence of antibodies against *T. gondii* by ELISA test (ID.vet Innovative Diagnostics, Grabels, France). Seropositive mice were killed at 42 days post-infection by chloroform-inhalation. Then, the mouse brain was homogenized with an equal volume of sterile normal saline by passing through a 16 g needle ten times by mean a syringe. One drop of given suspension placed on a slide and spread out covered with a slip and microscopically examined. At least five slides should be examined. The isolation of *T.gondii* was successful if *Toxoplasma* cysts were found in the mouse brain.

### PCR-RFLP analysis

PCR-RFLP with SAG1, SAG2, SAG3, BTUB and GRA6 markers were performed to determine the *T. gondii* genotype in fecal and brain samples according to described methods [8–11]. Briefly, the PCR reaction was performed in a 25 μl reaction mixture containing 1 μl of extracted DNA, 75 mM Tris-HCl (pH 8.5), 20 mM (NH4)2SO4, 1.5 mM MgCl2, 0.1% Tween 20, 0.2 mM dNTPs, 0.025 U/μl amplicon Taq DNA polymerase, inert red dye, a stabilizer and 10 pmol of each primer described in Table 1 After 5 min of initial denaturation at 95 °C, 35 cycles of amplification followed by 30 s at 94 °C, 1 min at 60 °C, 2 min at 72 °C, and a final extension of 72 °C for 10 min (MJ Mini Thermal Cycler, Bio-Rad Co, USA).

After that, 1.5 U of enzymes endonuclease with 2 U buffers was added to 15-mL of each PCR product and incubated as the manufacturer’s protocol. The digested products were electrophoresed to separate restriction fragments in 1.6% agarose gel. Finally, the agarose gel was stained with ethidium bromide and visualized under UV. The extracted DNA of the RH strain was used as a positive control.

### DNA sequencing

The purified PCR products of B1 with primers were sent to DNA sequencing in the Bioneer Inc. (Bioneer Company, Seoul, and Company). The assembling and editing of nucleotide sequences were used by CLc bio software.

### Statistical analysis

The chi-square test analyzed the relationship between infection rate and variables such as age and gender. A significant association was identified when a *p*-value of less than 0.05 was observed [44]. The agreement between the different tests was showed as a k-value. The agreement as poor if k-values between 0.2 and 0.4, moderate if k-values between 0.4 and 0.6, substantial if 0.6 and 0.8 and good if it exceeds 0.8 and 1.3 [44].

## Data Availability

The datasets used and/or analyzed (Persian language) during the current study are available from the corresponding author on reasonable request.

## Abbreviations

BTUB: Beta-tubulin
ELISA: Enzyme-linked immunosorbent assay
GRA-6: Dense granule protein-6
NCBI: National Center for Biotechnology Information
PCR: Polymerase chain reaction
PCR-RFLP: PCR-restriction fragment length polymorphism
SAG-1-3: Surface antigen-1-3
